# Olive Leaf Extract (OleaVita) Suppresses Inflammatory Cytokine Production and NLRP3 Inflammasomes in Human Placenta

**DOI:** 10.3390/nu11050970

**Published:** 2019-04-28

**Authors:** Yasuaki Kaneko, Michiya Sano, Kotomi Seno, Yuka Oogaki, Hironori Takahashi, Akihide Ohkuchi, Miki Yokozawa, Ken Yamauchi, Hisataka Iwata, Takehito Kuwayama, Koumei Shirasuna

**Affiliations:** 1 Laboratory of Animal Reproduction, Department of Animal Science, Tokyo University of Agriculture, Atsugi, Kanagawa 243-0034, Japan; 43517004@nodai.ac.jp (Y.K.); 43518008@nodai.ac.jp (M.S.); s11079@yahoo.co.jp (K.S.); 43519003@nodai.ac.jp (Y.O.); h1iwata@nodai.ac.jp (H.I.); takehito@nodai.ac.jp (T.K.); 2 Department of Obstetrics and Gynecology, Jichi Medical University, Shimotsuke, Tochigi 329-0498, Japan; hironori@jichi.ac.jp (H.T.); okuchi@jichi.ac.jp (A.O.); 3 Nutraceuticals Research Office, Nutrition Act Co., Ltd., Ginza, Chuo-ku, Tokyo 104-0061, Japan; m-yokozawa@n-act.co.jp (M.Y.); k-yamauchi@n-act.co.jp (K.Y.)

**Keywords:** placenta, interleukin, NLRP3 inflammasome, inflammation

## Abstract

The placenta is essential for pregnancy and produces both pro-inflammatory and anti-inflammatory cytokines. Excessive production of inflammatory cytokines, involving interleukin-1β (IL-1β), IL-6, and IL-8, from placental tissues is associated with pregnancy complications. Olive leaf extract has several health benefits, including anti-inflammatory functions. OleaVita is a new commercial olive leaf extract; it is hypothesized to suppress placental inflammation. In human placental tissue culture, OleaVita treatment inhibited the secretion of inflammatory cytokines and NF-κB p65 protein expression. OleaVita also suppressed toll-like receptor ligands-induced IL-1β secretion in human placental tissues. IL-1β is regulated by the NLRP3 inflammasomes, a pivotal regulator of various diseases. OleaVita significantly decreased NLRP3 and pro-IL-1β protein expression, suggesting that it has an inhibitory effect on NLRP3 inflammasome activation. Thus, OleaVita is beneficial as an inhibitor of inflammation and NLRP3 inflammasome activation, and may be used as a supplement for the treatment and prevention of inflammatory diseases.

## 1. Introduction

The placenta is an important organ for pregnancy, and produces both pro-inflammatory and anti-inflammatory cytokines, which regulate placental function and development [1]. Compared with the non-pregnant state, normal pregnancy is associated with increased systemic inflammation [2]. Alternatively, women with pregnancy complications, such as preterm, gestational diabetes, intrauterine growth restriction, and preeclampsia [3,4] develop accelerated systemic and placental inflammation [5]. Levels of pro-inflammatory cytokines such as interleukin (IL)-1β, IL-6, and IL-8 have been observed in the sera and placental tissues of pregnant women with these complications compared with those in healthy pregnant women [2,5,6,7]. However, because of concerns of toxicity to the fetus, pregnancy complications are very difficult to treat with drugs.

Inflammatory responses associated with gestational hypertension and preeclampsia can occur even in the absence of microbial infection. Thus, these syndromes are considered to be sterile inflammations [8,9]. Sterile inflammatory responses are mediated through nucleotide-binding oligomerization domain-like receptor (NLR) family pyrin domain-containing 3 (NLRP3) inflammasomes. NLRP3 inflammasomes are large multi-protein complexes in the cytosol that regulate the production of the pivotal inflammatory cytokine IL-1β [10,11]. NLRP3 inflammasomes are comprised of three different proteins: NLRP3; apoptosis-associated speck-like protein containing a caspase recruitment domain (ASC); and caspase-1 (an IL-1β-converting enzyme). NLRP3 inflammasomes are associated with various types of inflammatory diseases, such gout, cardiovascular diseases, metabolic syndrome, type 2 diabetes, and silicosis [10,11,12,13]. In the placenta and peripheral blood leukocytes of preeclamptic patients, the expression of NLRP3, caspase-1, and IL-1β is higher than in those of healthy pregnant women [14]. Pregnancy complications induced by angiotensin II, nanosilica, and extracellular vesicles are improved in NLRP3-deficient mice since the suppression of placental inflammation [9,15,16]. These findings suggest that the inhibition of NLRP3 inflammasomes is essential to counter pregnancy dysfunctions.

The olive, *Olea europaea*, grows in the Mediterranean regions, Asia, and Africa, and is one of the most relevant traditional drugs. Olive leaf extracts is known to be a natural resource of various beneficial substances, such as polyphenols, oleuropein, and oleanolic acid, and have been used for various effects, including anti-inflammatory, anti-hypertensive, and hypocholesterolemic activities [17,18]. There are many reports stating that oleanolic acid has an anti-inflammatory effect both in vivo and in vitro [19,20,21,22]. Recently, a new product, an ethanol/water extract of olive leaves (OleaVita^®^, Phytodia S.A.S., Illkirch-Graffenstaden, France), has been developed [23]. This extract contains large amounts of oleanolic acid, which activates TGR5 (G protein-coupled bile acid receptor 1) and is reported to have an obesity-suppressing effect in humans [23]. However, to the best of our knowledge, there have been no investigations into the effect of olive leaf extracts, including OleaVita, on human placental function.

In the present study, we assess the role of the olive leaf extract OleaVita in suppressing inflammatory cytokine production and in NLRP3 inflammasome activation in human placenta. To test our hypothesis, we investigated whether OleaVita has an inflammatory inhibitory effect and what mechanism controls inflammation using in vitro human placental tissue culture model.

## 2. Materials and Methods

### 2.1. Olive Leaf Extract (OleaVita)

OleaVita^®^, an ethanol/water extract of olive leaves, was used in the present study. It was obtained from Phytodia S.A.S. (Illkirch-Graffenstaden, France). A powdered extract of OleaVita was dissolved in dimethylsulfoxide (DMSO) at 2 g/mL as a stock solution.

### 2.2. Tissue Collection

Human placentae were obtained from a total of twelve women, who delivered healthy, singleton infants at term (patients who gave informed consent were obtained using protocols approved by the Jichi Medical University Ethics Committee). The relevant clinical details of the patients are shown in Table 1. In the present study, we used 4–5 placentas in each experiment and totally we used 12 placentas (9 placenta of females and 3 placenta of males). As shown in our previous study [24], tissues were obtained within 20 min of delivery and dissected fragments were placed in ice-cold phosphate buffered saline (PBS). Placental tissue was blunt dissected to remove visible connective tissue and cut into small pieces (about 50 mg wet weight). These placental tissue pieces were placed on a cell culture insert membrane (0.4 µm membrane, 24-well plate, Thermo Fisher Scientific, Inc., Waltham, MA, USA) with 1 mL Dulbecco’s modified Eagle’s medium/F-12 (DMEM/F-12; Life Technologies Corporation, Carlsbad, CA, USA) supplemented with antibiotics including amphotericin B and gentamicin (Sigma-Aldrich, St. Louis, MO, USA), and 5% fetal calf serum (FCS, ICN Pharmaceuticals, Inc., Costa Mesa, CA, USA).

### 2.3. Experimental Conditions

Experiment 1: Human placental tissues were incubated with control or OleaVita (0.1 or 1 mg/mL) for 6 h at 37 °C. In preliminary experiment, we checked the appreciate dose of OleaVita (0.01, 0.1, 1, or 2 mg/mL) and incubation time (6 or 24 h). Supernatant and tissues were collected for enzyme-linked immunosorbent assay (ELISA), western blotting, and real-time Reverse transcription polymerase chain reaction (RT-PCR), and were stored at −20 °C or −80 °C before use.

Experiment 2: To investigate the role of Jun N-terminal kinase (JNK), human placental tissues were incubated with OleaVita (1 mg/mL) with or without SP600125 (a JNK inhibitor, 10 μM, Merck Millipore, Burlington, MA, USA), for 6 h at 37 °C. Supernatant and tissues were collected for ELISA and were stored at −20 °C before use.

Experiment 3: To investigate the role of OleaVita on toll-like receptor (TLR) ligand-induced inflammatory responses, human placental tissues were pre-incubated with or without OleaVita (1 mg/mL) for 1 h. Tissues were then incubated with zymosan (TLR2 agonist, 50 μM, Invivogen, Carlsbad, CA, USA), lipopolysaccharide (LPS, TLR4 agonist, 1 μg/mL, Sigma-Aldrich), or imiquimod (TLR7 agonist, 20 μM, Invivogen) for 6 h at 37 °C. Supernatant and tissues were collected for ELISA and were stored at −20 °C before use.

### 2.4. Determination of Cytokines

Levels of IL-1β, IL-6, or IL-8 were determined using a human ELISA kit (R&D Systems, Minneapolis, MN, USA) according to the manufacturer’s instructions.

### 2.5. Real-Time RT-PCR

Total RNA was prepared using ISOGEN II (Nippon Gene Company, Limited, Toyama, Japan) according to the manufacturer’s instructions. RNA extraction and cDNA production were performed using a commercial kit (ReverTra Ace; Toyobo Co., Ltd., Osaka, Japan). Real-time RT-PCR was performed using the CFX Connect^TM^ Real Time PCR (Bio-Rad, Hercules, CA, USA) and a commercial kit (Thunderbird SYBR qPCR Mix; Toyobo Co., Ltd.) to detect mRNA expressions of *IL-1β*, *IL-6*, *IL-8*, *Caspase-3*, or *glyceraldehyde 3-phosphate dehydrogenase (GAPDH)*. The following antisense and sense primers were used: *IL-1β* (5′- TGATGGCTTATTACAGTGGCAATG-3′ and 5′- GTAGTGGTGGTGGGAGATTCG-3′, NM-000576), *IL-6* (5′- AAATTCGGTACATCCTCGACGG -3′ and 5′- GGAAGGTTCAGGTTGTTTTCTGC-3′, M54894), *IL-8* (5′- CTTGGCAGCCTTCCTGATTTC -3′ and 5′- GGTGGAAAGGTTTGGAGTATGTCT -3′, BC013615.1), *Caspase-3* (5′- CATGCAAGCGAATCAATGGACT -3′ and 5′- CTGTACCAGACCGAGATGTCA -3′, AJ413269.1), and *GAPDH* (5′- AAATGAGCCCCAGCCTTCT-3′ and 5′- AGGATGTCAGCGGGAGCCGG-3′, M33197). RT-qPCR was performed in duplicate with a final reaction volume of 20 µL containing 10 µL SYBR Green, 7.8 µL distilled water, 0.1 µL 100 µM forward and reverse primers, and 2 µL of cDNA template. The amplification program consisted of a 5 min denaturation at 95 °C followed by 40 cycles of amplification (95 °C for 15 s, 60 °C for 30 s, and 72 °C for 20 s). Expression levels of each target gene were normalized to corresponding GAPDH threshold cycle (CT) values using the ΔΔ CT comparative method [25].

### 2.6. Determination of Cytokines and Lactate Dehydrogenase (LDH)

After incubation, supernatant were collected and stored at −20 °C before use. Levels of LDH as a cell death marker was determined using cytotoxicity detection kit (Roche Diagnostics GmbH, Mannheim, Germany).

### 2.7. Western Blot Analysis

Lysates from the placental tissues were prepared using RIPA buffer (Wako Pure Chemical Industries, Osaka, Japan). Placental tissues were washed with cold PBS, minced and incubated with RIPA buffer for 15 min on ice into 1.5 mL tubes. Placental tissue lysates were subsequently centrifuged at 12,000× *g* for 20 min at 4 °C. Supernatants were transferred to a fresh tube and stored at −80 °C before analysis. A total of 10 µg protein was loaded per lane and separated by 10% SDS-PAGE. The expression of protein was analyzed by western blot. After transfer onto polyvinylidene fluoride membranes, nonspecific antibody binding was blocked for 1 h at room temperature using Immunoblock (DS Pharma Biomedical Co, Ltd, Osaka, Japan). Then, membranes were incubated for 24 h at 4 °C with anti-NF-κB p65 antibody (1:500, EMD Millipore Corporation, Temecula, CA, USA), anti-phospho-IκB antibody (1:500, R&D systems), anti-IκB antibody (1:500, R&D systems), anti-IL-1β antibody (1:1000, Santa Cruz Biotechnology, Dallas, TX, USA), anti-NLRP3 antibody (1:250, R&D systems), anti-ASC antibody (1:1000, Santa Cruz Biotechnology, Santa Cruz, CA, USA), anti-phospho-c-Jun antibody (1:1000, Cell Signaling Technology, Danvers, MA, USA), anti-c-Jun antibody (1:1000, Cell Signaling Technology),and anti-β-actin (ACTB) antibody (1:10000, Sigma-Aldrich), followed by an incubation for 1 h with secondary antibody conjugated horseradish peroxidase (HRP; 1:1000, GE Healthcare, UK Ltd, Buckinghamshire, UK). Immunoreactive bands were visualized by Western BLoT Quant HRP Substrate (GE Healthcare) using ImageQuant LAS 4000 (GE Healthcare). The results represent at least 3 independent experiments. Quantitative analysis of bands was performed using Image J (National Institutes of Health, Bethesda, MD, USA).

### 2.8. Statistical Analysis

Data are expressed as mean ± standard error of the mean (SEM). Differences between treatment groups were identified nonparametric analysis of variance, followed by Mann–Whitney U-test or Kruskal test. A *p*-value of <0.05 was considered statistically significant.

## 3. Results

### 3.1. Effects of OleaVita on Inflammatory Cytokine Production in Human Placental Tissues

**Figure 1:**
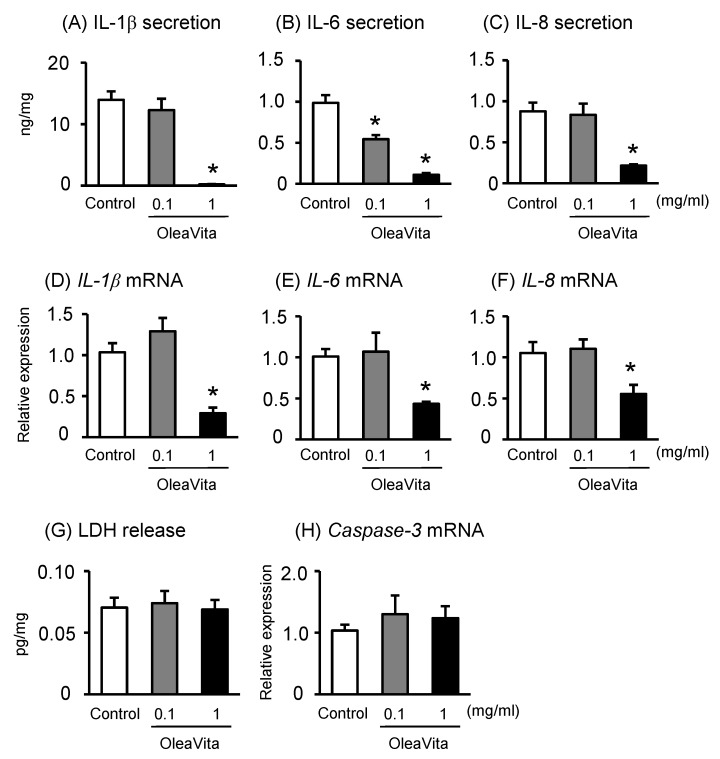
Effects of OleaVita on inflammatory cytokine production in human placental tissues. Human placental tissues were incubated for 6 h with OleaVita (0.1 or 1 mg/mL). After incubation, IL-1β (**A**), IL-6 (**B**), and IL-8 (**C**) levels in the supernatant were determined using ELISA. The IL-1β, NLRP3, ASC, CASP1, and Caspase-3 mRNA levels were measured using qRT-PCR (**D**–**F**, and **H**). The release of LDH levels were subsequently quantified in the supernatant (**G**). Data are expressed as means ± SEM (*n* = 5, 3 female and 2 male placentas). Differences between treatment groups were identified nonparametric analysis of variance, followed by a Kruskal test; * *p* < 0.05.

We examined the effects of OleaVita on the regulation of inflammatory cytokine production using human placental tissue cultures. The secretion of the cytokines IL-1β, IL-6, and IL-8 in human placental tissue culture were significantly decreased following OleaVita treatment (Figure 1A–C). Similar with cytokine secretions, the expression of the corresponding mRNAs was also decreased after OleaVita treatment (Figure 1D–F). On the other hand, treatment with OleaVita did not affect the levels of LDH (as a cell death marker) and mRNA expression of caspase-3 (as an apoptosis marker), suggesting that OleaVita has no toxic effects on human placenta (Figure 1G,H). In this experiment, we used three placentas of females and two placentas of males and results indicated that the response to OleaVita did not change between placentas from females and males (Figure S1).

### 3.2. Effects of OleaVita on the NF-κB Pathway in Human Placental Tissues

**Figure 2:**
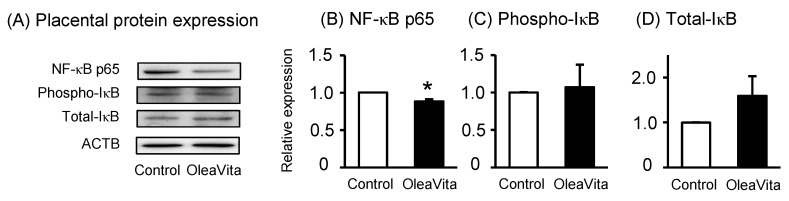
Effects of OleaVita on the NF-κB pathway in human placental tissues. Human placental tissues were incubated for 6 h with OleaVita (1 mg/mL). After incubation, NF-κB p65, phospho-IκB, total-IκB, and ACTB protein levels were subsequently quantified in the cell lysates by western blot (**A**–**D**). Data are expressed as means ± SEM (*n* = 4, 2 female and 2 male placentas). Differences between treatment groups were identified nonparametric analysis of variance, followed by the Mann–Whitney *U*-test; * *p* < 0.05.

It is well known that NF-κB is a key transcription factor for the production of inflammatory cytokines [26]. Transcriptional activity of NF-κB is dependent on IκB phosphorylation as an NF-κB inhibitor. After IκB phosphorylation, the NF-κB p65 subunit is transported into the nucleus, where it promotes the expression of inflammatory cytokines. Although there was no observed effect on phospho-IκB and total-IκB protein expression, treatment with OleaVita considerably reduced NF-κB p65 protein expression in human placental tissue culture (Figure 2A–D).

### 3.3. Effects of OleaVita on NLRP3 Inflammasomes in Human Placental Tissues

**Figure 3:**
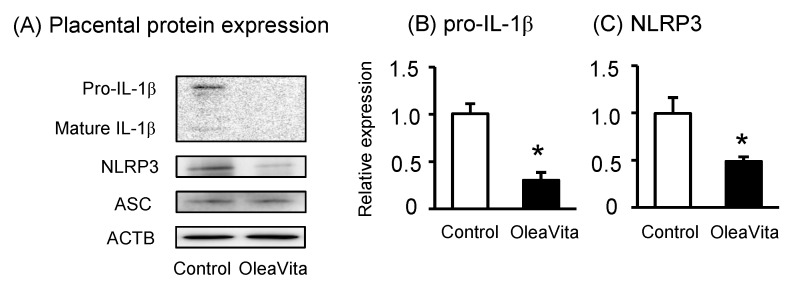
Effects of OleaVita on NLRP3 inflammasomes in human placental tissues. Human placental tissues were incubated for 6 h with OleaVita (1 mg/mL). After incubation, IL-1β, NLRP3, ASC, and ACTB protein levels were subsequently quantified in the cell lysates by western blot (**A**–**C**). Data are expressed as means ± SEM (*n* = 4, 2 female and 2 male placentas). Differences between treatment groups were identified nonparametric analysis of variance, followed by the Mann–Whitney *U*-test; * *p* < 0.05.

Because OleaVita clearly suppressed IL-1β secretion from human placental tissue, we assessed the potential role of OleaVita in inhibiting NLRP3 inflammasome activation. As expected, OleaVita treatment significantly decreased pro-IL-1β and NLRP3 protein expression in human placental tissues (Figure 3A–C). OleaVita treatment significantly decreased the expression of mRNA for pro-IL-1β and NLRP3 in human placental tissues [27]. Conversely, treatment with OleaVita did not affect ASC protein expression (Figure 3A). These findings suggest that OleaVita inhibits mature IL-1β secretion via the down-regulation of both pro-IL-1β and NLRP3 inflammasomes in human placenta.

### 3.4. Effects of OleaVita on the JNK Pathway in Human Placental Tissues

**Figure 4:**
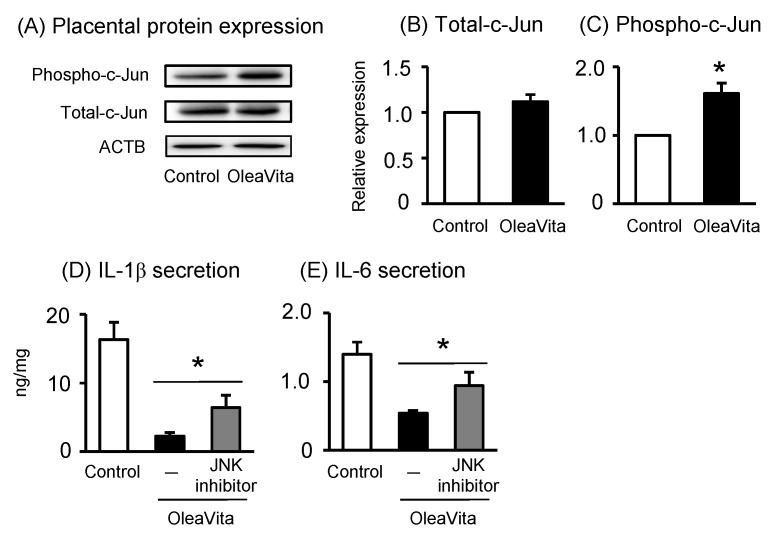
Effects of OleaVita on the JNK pathway in human placental tissues. Human placental tissues were incubated for 6 h with OleaVita (1 mg/mL). After incubation, phosphor-c-Jun, c-Jun, and ACTB protein levels were subsequently quantified in the cell lysates by western blot (**A**–**C**). Tissues were pre-incubated for 1 h with specific pharmacological inhibitors (SP600125 as a JNK inhibitor). After inhibitor treatments, tissues were incubated for 6 h with Oleavita (1 mg/mL). IL-1β and IL-6 levels in the supernatant were determined using ELISA (**D** and **E**). Data are expressed as means ± SEM (*n* = 4, 3 female and 1 male placentas). Differences between treatment groups were identified nonparametric analysis of variance, followed by the Mann–Whitney *U*-test; * *p* < 0.05.

It has been reported that oleanolic acid, one of the major components of Oleavita, activates the c-Jun-N-terminal kinase (JNK) pathway, resulting in the regulation of inflammatory cytokines [28]. JNK also phosphorylates and activates the transcriptional factor c-Jun, one of the major molecules involved in JNK signaling. Therefore, we investigated the role of the JNK pathway in mediating the effects of OleaVita in human placenta. OleaVita treatment remarkably increased the expression of phosphorylated c-Jun protein, but not of total c-Jun protein, in human placental tissues (Figure 4A–C). To determine the importance of the JNK pathway, placental tissues were incubated with JNK inhibitor (SP600125) before treatment with OleaVita. Although treatment with OleaVita clearly decreased the secretion of IL-1β and IL-6, JNK inhibitor considerably attenuated the inflammation suppressing effect of OleaVita (Figure 4D,E). These findings indicate that the activation of the JNK pathway is necessary for the inhibitory effect of OleaVita on inflammatory cytokine production in human placenta.

### 3.5. Effects of OleaVita on TLR Ligands-Induced IL-1β Secretion in Human Placental Tissues

**Figure 5:**
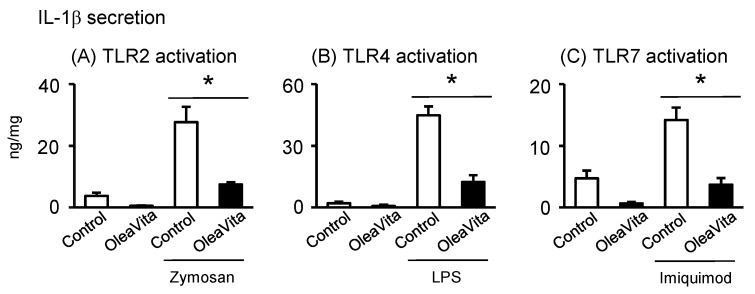
Effects of OleaVita on TLR ligands-induced IL-1β secretion in human placental tissues. Human placental tissues were pre-incubated for 1 h with OleaVita (1 mg/mL). After incubation, tissues were incubated for 6 h with each TLR-ligands (zymosan as TLR2 ligand, LPS as TLR4 ligand, and Imiquimod as TLR7 ligand). After inhibitor treatments, IL-1β levels in the supernatant were determined using ELISA (**A**–**C**). Data are expressed as means ± SEM (*n* = 4, all female placentas). Differences between treatment groups were identified nonparametric analysis of variance, followed by the Mann–Whitney U-test; * *p* < 0.05.

Finally, we investigated whether excessive inflammation of the TLR ligand signal can be reduced by OleaVita, and whether there is selectivity in the anti-inflammatory effects of OleaVita in human placenta. In our placental tissue culture model, three TLR ligands, including zymosan (TLR2), LPS (TLR4), and imiquimod (TLR7), remarkably stimulated IL-1β secretion in human placental tissues (Figure 5A–C). However, two TLR ligands, poly(I:C) (TLR3) and CpG ODN (TLR9), did not stimulate IL-1β secretion (data not shown). Importantly, treatment with OleaVita dramatically suppressed the secretion of IL-1β induced by the three TLR ligands in human placental tissues (Figure 5A–C). Thus, our findings indicated that OleaVita has a role in suppressing excessive inflammatory responses, independent of TLR ligands, in human placenta.

## 4. Discussion

Increasing evidence indicates that olive leaf extracts have various beneficial effects, such as the inhibition of inflammation, reduction of oxidative stress, and amelioration of apoptosis in the kidney, liver, aorta, and intestine [17,18]. However, the role of olive leaf extract, including OleaVita, in human placental tissues has not been understood until now. In the present study, we showed that OleaVita treatment directly inhibited the secretion of inflammatory cytokines, including IL-1β, IL-6, and IL-8, with a decrease in the expression of their mRNA via suppression of the NF-κB transcription pathway in human placental tissues. OleaVita has already been used in humans (It is a standard of about 1 g daily for supplements) and any toxic effects have not been noted (No toxicity after continuous administration of 2 g/kg/day for 28 days in rats). Therefore, similar to other olive leaf extracts, OleaVita has the potential to be used as a supplement for the prevention and treatment of inflammatory diseases, involving pregnancy complications, in humans. Before applying to human pregnancy, it is necessary to examine the safety of experimental animals during pregnancy.

OleaVita has been found to have an effect which is agonistic to that of TGR5 [23]. It has been reported that overexpression of TGR5 in THP-1 cells (a human monocyte cell line) decreases the production of LPS-stimulated cytokine [29]. Oleanolic acid, one of the major components of OleaVita, is known to be a TGR5 agonist, and oleanolic acid activates JNK via TGR5, resulting in the regulation of inflammatory cytokine production and a reduction in obesity [28,30]. In the present study, treatment with OleaVita activated c-Jun downstream from the JNK pathway, and treatment with a JNK inhibitor attenuated the inflammation suppressing effect of OleaVita. Therefore, it is considered that the inflammation suppressing effect of OleaVita may be mediated through the TGR5-JNK-NF-κB pathway in human placenta.

The NLRP3 inflammasomes have emerged as a key regulator of various types of chronic and noninfectious inflammatory responses and diseases [10,11]. Importantly, we demonstrated that in human placental tissues, OleaVita notably inhibited the activation of NLRP3 inflammasomes, resulting in a decrease in the secretion of mature IL-1β. Consistent with our present results, An et al. [31] and Kim et al. [19] reported that oleanolic acid attenuated carotid artery and pulmonary injuries through the inhibition of NLRP3 inflammasomes using in vivo experimental models. Recently, NLRP3 inflammasome activation has been shown to be involved in various pregnancy complications, such as preeclampsia, spontaneous preterm labor, and hypertension in pregnancy [9,14]. Thus, OleaVita, which has potential as a supplementation for the inhibition of NLRP3 inflammasomes, may lead to the alleviation of various types of pregnancy complications and non-communicative disorders in humans. In the future, it will be important to examine how OleaVita exerts its effects in specific disease models and human beings.

Because the OleaVita used in the present study is an extract of olive leaves and includes a range of components, it is difficult to identify the components responsible for the effect of OleaVita. Among various these components, oleanolic acid is one of the main components of OleaVita [23]; and oleanolic acid has been found to have strong anti-inflammatory effect and improves pathological condition. For example, oleanolic acid treatment has been shown to alleviate lung injury in mice, including the inhibition of immune cell accumulation and inflammatory cytokine production [19]. Djeziri et al. [20] reported that oleanolic acid exerts protective effects on obesity, including a decrease in body and adipose tissue weight and inflammatory cytokine production. In the reproductive experimental field, Zhao et al. [32] showed that oleanolic acid effectively rejuvenated testicular function by reducing DNA damage, apoptosis, and inflammatory cytokine production, indicating a potential for the prevention of aging-related testicular dysfunction. On the other hand, as OleaVita contains many other components, such as oleuropein and polyphenolic compounds, further studies are necessary warranted to understand the exacts effect of OleaVita.

Mitochondria are one of the targets of olive leaf extracts such as OleaVita. Indeed, OleaVita treatment increased mRNA expression of PGC1α, which promotes the synthesis of mitochondria in 3T3-L1 adipocytes [23]. In addition, oleanolic acid mitigates the dysfunction of mitochondrial membrane potential and ATP production induced by high glucose levels in chondrocytes [22]. In the present study, treatment with OleaVita increased mRNA expression of PGC1α and factors regulating mitochondrial fusion (mitofusion (MFN) 1 and 2 and optic atrophy 1 (OPA1)) and division (dynamin-related protein 1 (DRP1)) in human placental tissues (Figure S2), suggesting a potential role for OleaVita in the regulation of mitochondrial biogenesis. However, there was no effect on mitochondrial copy number, probably because of the short incubation time (6 h) used in the present study. To determine the effect of OleaVita on mitochondrial function, further investigation is needed, using placental cells rather than placental tissues.

## 5. Conclusions

In conclusion, we demonstrated that exposure to OleaVita results in the suppression of inflammatory cytokine production and the activation of NLRP3 inflammasomes, and TLR-ligands induced inflammatory responses in human placenta. In addition, JNK/NF-κB signaling plays a pivotal role in the inhibitory effect of OleaVita on inflammatory responses. Further insights into the exact role of OleaVita in obesity, placental inflammation, and pregnancy complications will be useful for developing preventive and therapeutic strategies for these complications.

## Figures and Tables

**Table 1:** Individual characteristics.

Variable	Normal Pregnancy (*n* = 12)
Age of mother	35.4 (27–40)
Maximum systolic blood pressure (mmHg)	112.3 (88–132)
Maximum diastolic blood pressure (mmHg)	70.8 (48–96)
Delivery gestation (week)	38.0 (37–39)
Placental weight (g)	540.3 (415–627)
Birthweight (g)	2712.7 (2066–3660)

Data represented as median (range).

## Supplementary Materials

The following are available online at https://www.mdpi.com/2072-6643/11/5/970/s1, Figure S1: Effects of OleaVita on IL-1β secretion depending on the sex in human placental tissues, Figure S2: Effects of OleaVita on mitochondria in human placental tissues.

## Abbreviations

ASC	apoptosis-associated speck-like protein containing a caspase recruitment domain
DRP1	dynamin-related protein 1
FCS	fetal calf serum
GAPDH	glyceraldehyde 3-phosphate dehydrogenase
IL	interleukin
JNK	Jun N-terminal kinase
LDH	lactate dehydrogenase
LPS	lipopolysaccharide
MFN	mitofusion
NLR	nucleotide-binding oligomerization domain-like receptor
NLRP3	NLR family pyrin domain-containing 3
OPA1	optic atrophy 1
TGR5	G protein-coupled bile acid receptor 1
TLR	toll-like receptor ligand
