# Supplementary material for: Olive Leaf Extract (OleaVita) Suppresses Inflammatory Cytokine Production and NLRP3 Inflammasomes in Human Placenta
Source: Nutrients. 2019 Apr 28;11(5):970. doi: 10.3390/nu11050970 (PMC6566934; doi:10.3390/nu11050970)

Olive leaf extract (Oleavita) suppresses inflammatory cytokine production and NLRP3 inflammasomes in human placenta

Yasuaki Kaneko[1#], Michiya Sano[1#], Kotomi Seno[1], Yuka Oogaki[1], Hironori Takahashi[2], Akihide Ohkuchi[2], Miki Yokozawa[3], Ken Yamauchi[3], Hisataka Iwata[1], Takehito Kuwayama[1], Koumei Shirasuna[1*]

Supplementary Data

Materials and methods

Copy number of Mitochondrial DNA

Human placentae were obtained from a total of five women and extracted mitochondrial DNA using a mtDNA Extractor CT kit (Nippon Gene Company, Limited, Toyama, Japan) according to the manufacturer's instructions. Then, mitochondrial copy number was determined using a Human Mitochondrial DNA monitoring primer set (Takara, Shiga, Japan) according to the manufacturer's instructions using the CFX Connect™ Real Time PCR (Bio-Rad, Hercules, CA).

Real-time RT-PCR for mitochondrial function-related genes

Total RNA, RNA extraction and cDNA production were performed as described method. Real-time RT-PCR was performed using the CFX Connect™ Real Time PCR and a commercial kit to detect mRNA expressions of *PGC1a*, *mitofusion (MFN) 1, MFN2, optic atrophy 1 (OPA1), dynamin-related protein 1 (DRP1), superoxide dismutase (SOD) 1,* or *SOD2*. The following antisense and sense primers were used: *PGC1a* (5′- CTCAAATATCTGACCACAAACGATGACCCTC -3′ and 5′- GTTGTTGGTTTGGCTTGTAAGTGTTGTGAC -3′), *MFN1* (5′-

TGTTTTGGTCGCAAACTCTG -3′ and 5′- CTGTCTGCGTACGTCTTCCA -3′), *MFN2* (5′- AGCTGGACAGCTGGATTGAC -3′ and 5′- GCTTTTCCGTCTGCATCAGG -3′), *OPA1* (5′- TGCCTGACATTGTGTGGGAA -3′ and 5′- CTTCCGGAGAACCTGAGGTAA -3′), *DRP1* (5′- TGCTTCCCAGAGGTACTGGA -3′ and 5′- TCTGCTTCCACCCCATTTTCT -3′), *SOD1* (5′- AATGGACCAGTGAAGGTGTGGGG -3′ and 5′- CACATTGCCCAAGTCTCCAACA -3′),and *SOD2* (5′- ATGTTGAGCCGGGCAGTGTG-3′ and 5′- GTGCAGCTGCATGATCTGCG-3′). The amplification program consisted of a 5 min denaturation at 95°C followed by 40 cycles of amplification (95°C for 15 sec, 60°C for 30 sec, and 72°C for 20 sec). Expression levels of each target gene were normalized to corresponding GAPDH threshold cycle (CT) values using the ΔΔ CT comparative method [25].

Results

Effects of OleaVita on IL-1β secretion depending on the sex in human placental tissues

We examined the difference of placental sex for effects of OleaVita using human placental tissue cultures. As shown in Sup. Figure 1, OleaVita inhibited IL-1β in the same manner in both male and female placentas, indicating the no sex dependent effect on placenta in the present study.

Supplementary Figure 1

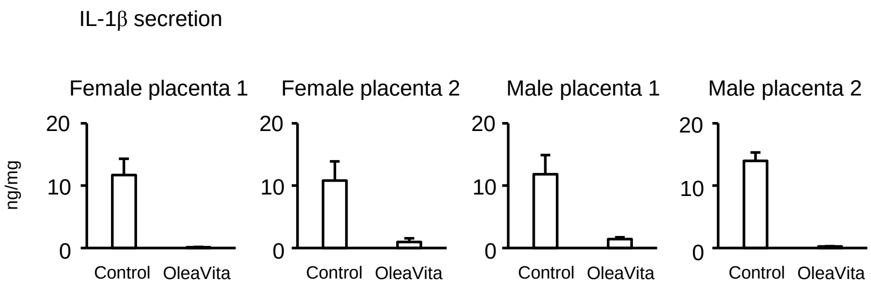

Effects of OleaVita on mitochondria in human placental tissues

We examined the effects of OleaVita on mitochondrial function using healthy human placental tissue cultures. Mitochondrial DNA copy number did not affect by treatment with OleaVita

(Sup. Fig. 2A). Treatment with OleaVita significantly mRNA expression of PGC1a, MFN1, OPA1, and DRP1, whereas mRNA expression of MFN2, SOD1, and SOD2 were up-regulated (not significant) by treatment with OleaVita in human placental tissues (Sup. Fig. 2B-H).

Supplementary Figure 2

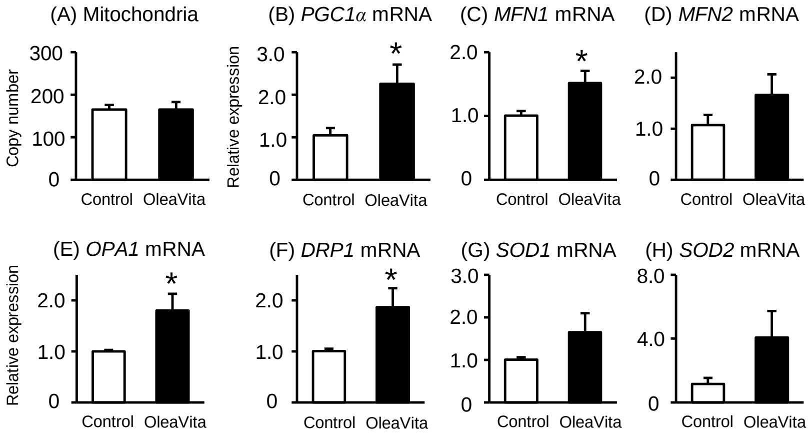

Supplement: Supplementary file 1 [file nutrients-11-00970-s001.pdf]
